# Characterization by SEM, TEM and Quantum-Chemical Simulations of the Spherical Carbon with Nitrogen (SCN) Active Carbon Produced by Thermal Decomposition of Poly(vinylpyridine-divinylbenzene) Copolymer

**DOI:** 10.3390/ma2031239

**Published:** 2009-09-07

**Authors:** Volodymyr D. Khavryuchenko, Oleksiy V. Khavryuchenko, Andriy I. Shkilnyy, Denys A. Stratiichuk, Vladyslav V. Lisnyak

**Affiliations:** 1 Institute for Sorption and Problems of Endoecology, National Academy of Sciences of Ukraine / 13 General Naumov str., UA-03167, Kyiv, Ukraine; E-Mail: vkhavr@rumbler.ru; 2 Chemical Department, Kyiv Taras Shevchenko National University / 64 Volodymyrska str., UA-01033, Kyiv, Ukraine; E-Mail: andriy.shkilnyy@gmail.com; 3 Institute for Superhard Materials, National Academy of Science of Ukraine / 2 Avtozavodska str., UA-04074, Kyiv, Ukraine; E-Mail: strat1@yandex.ru

**Keywords:** active carbon, carbonization, electron microscopy (SEM, TEM), energy dispersive X-ray analysis (EDX), computer modeling and simulation

## Abstract

Amorphous Spherical Carbon with Nitrogen (SCN) active carbon has been prepared by carbonization of poly(vinylpyridine-divinylbenzene) (PVPDVB) copolymer. The PVPDVB dehydrogenation copolymer has been quantum chemically (QC) simulated using cluster and periodic models. Scanning electron microscopy (SEM), transmission electron microscopy (TEM) and energy dispersive X-ray (EDX) studies of the resulting product have conformed the QC computation results. Great structural similarity is found both at the nano- and micro-levels between the N-doped SCN carbon and its pure carbonic SKS analog.

## 1. Introduction

Active and activated carbons (AC) doped with heteroatoms form a separate subclass of carbon materials (CMs) with exclusive absorptive and catalytic characteristics [1,2,3,4,5,6,7]. CMs can be doped with oxygen, boron, phosphorous, sulfur *etc*., but the most widespread ones are the nitrogen-doped (N-doped) carbons. Generally, the N-doped CMs are produced by activation of the neat carbon material with a N-containing modifier (*e*.*g*., NH_3_, urea or melamine) and successive thermal treatment [1,2,8,9,10,11], or by direct carbonization of the N-containing organic precursor [1,2,7,12,13]. In the latter case the resulting N-doped AC often shares the same features with the pure carbon-based ACs.

The Spherical Carbon with Nitrogen (SCN) AC produced by thermal degradation of poly(vinylpyridine-divinylbenzene) (PVPDVB) copolymers is one of the widely used N-doped CMs [7]. It is natively related with the SKS pure AC, similarly produced from polystyrene-divinylbenzene copolymer [14,15]. Acid/base, red/ox and sorption properties are more pronounced for the SCN AC, presumably due to the effect of N-doping [1,16,17,18]. In order to explain their origin one needs to characterize the macroscopic properties and to evaluate an atomic level model of the SCN AC, comparing them with previously described SKS-type ones [19].

However, examination of the SCN AC shares the same experimental problems with the SKS, since the principles of the CM structure are still uncertain. The experimental examination of the CM on an atomic level is hindered by a variety of factors, namely, the CM amorphicity, high absorption of electromagnetic radiation and non-uniform character of chemically active groups. The current theory of the CM structure postulates that it consists of two domains, namely, 1) the graphite-like particles and 2) the amorphous part [20,21]. N-atoms might be present in both the graphite-like and amorphous domains of the SCN, altering their electronic and spatial structure and thus affecting their physical and chemical properties. The impact of N-doping on the graphite-like domain has been studied recently in [22], where a minor change of structural and electronic characteristics as the N content increased was shown. In order to comprehensively characterize the SCN, the properties of the amorphous domain should also be described.

Electronic microscopy methods provide an opportunity to directly examine the microstructure of complex objects. These techniques were widely used for characterization of CMs earlier [1,2,19], so the SCN AC was studied in the current paper by SEM and TEM. However, instrumental characterization of the SCN nanostructure on atomic level is problematic, so indirect ways should also be used. The quantum chemical (QC) simulation of the PVPDVB high-temperature dehydrogenation should propose the model of the amorphous domain of the SCN AC, analogously to the SKS [19]. The purpose of the paper was to check whether the structural characteristics of SKS and SCN differ at the micro- and nano-levels.

## 2. Results and Discussion

**Figure 1:**
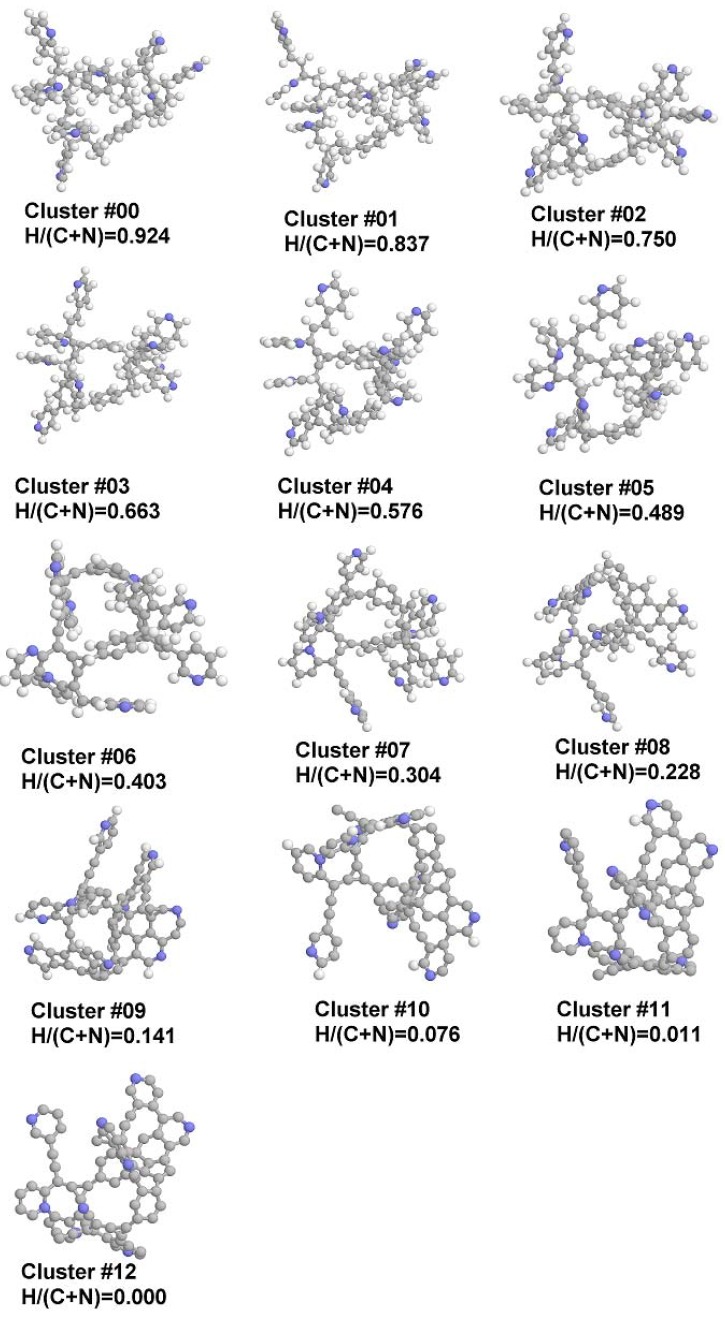
Structures of studied clusters (point number) and H/(C + N) relationship.

The PVPDVB copolymer has been simulated by the cluster shown in Figure 1 (hereinafter cluster C#0 or starting cluster) constructed from four 4-vinylpyridine, four 3-vinylpyridine, one 2-vinylpyridine and two 1,4-divinylbenzene residues. Such a ratio of vinylpyridine isomers (4:4:1) agrees with experimental data on the composition of the commercially used monomer mixture. The cluster has been constructed as a closed-loop, without termination of the “free” bonds at the ends of the polymer chains, and contains 83 carbon and nine nitrogen atoms. Then, according to the “technology following” methodology [23,24] the even number of hydrogen atoms has been removed (except for the cluster #12, where the only one last proton is eliminated) and the geometry of the cluster has been optimized. The procedure has been continued until complete elimination of the hydrogen from the cluster, resulting in 12 structures, denoted as clusters C#*X*, where *X* ranges from 1 to 12 and corresponds to the level of the dehydrogenation (cluster #12 contains an odd number of electrons and thus was simulated in ^2^*D* spin state). The degree of the carbonization of the cluster is characterized by the H/(C + N) ratio.

**Figure 2:**
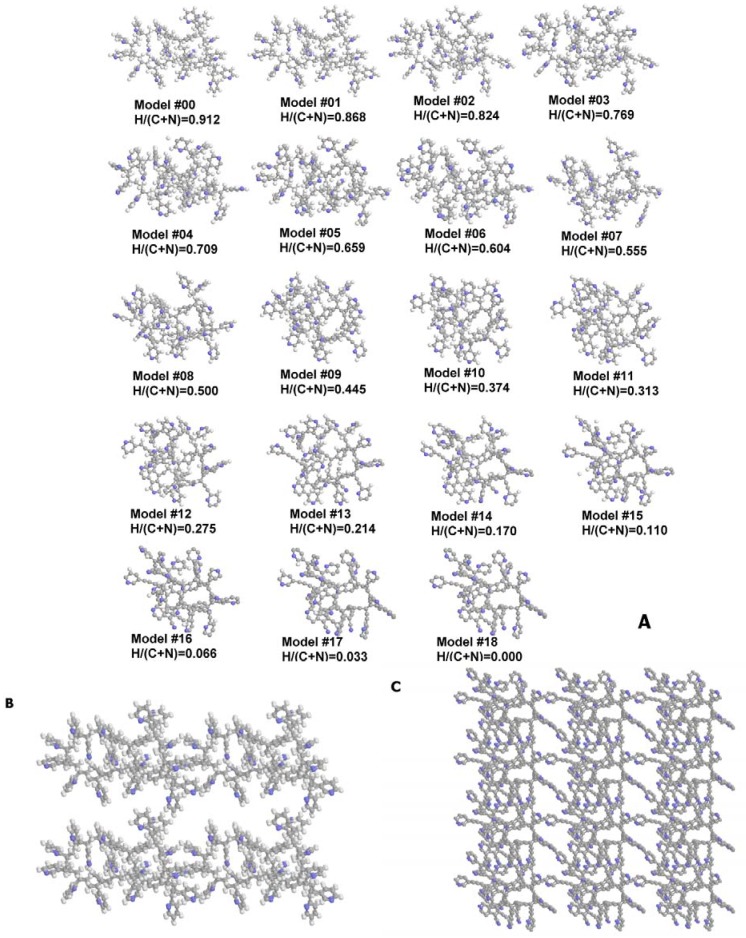
(a) Structures of studied periodic models basic units (point number) and H/(C + N) relationship. (b) starting periodic model (M#00) 2 × 2 × 2 supercell. (c) final periodic model (M#18) 2 × 3 × 4 supercell.

The periodic model has been constructed from the closed-loop clusters of four 5-vinylpyridine, eight 3-vinylpyridine, seven 2-vinylpyridine and two 1,4-divinylbenzene residues, translated in three dimensions in a cell with edges 33.0 Å, 24.4 Å and 21.27 Å (hereinafter model M#00 or starting model). The QC optimization has been performed under cyclic boundary conditions, including both optimization of atoms in the cell and optimization of the cell dimensions. Dehydrogenation has been simulated analogously to the cluster model (see above), resulting in 18 models, denoted as periodic models M#*Y*, where *Y* ranges from 1 to 18 and corresponds to the level of the dehydrogenation (Figure 2).

The sequence of hydrogen atoms’ elimination has been defined based on the literature data [25] showing that the aliphatic H atoms are more mobile compared to the aromatic ones. Therefore, the *sp*^3^-hybridized carbon atoms have been dehydrogenated first, and then the *sp*^2^-hybridized carbon atoms have been dehydrogenated. The sequence of the elimination within each type of H atoms has been chosen randomly.

The stages of mechanical transformations of the copolymer under heating were omitted due to the time-consuming computations. Therefore, the models are the first approximation level.

The following statistical structural characteristics have been evaluated from the QC simulated clusters and periodic models: (1) C and N coordination number distribution and (2) cycles size distribution.

### 2.1. Characterization on Micro-Level

**Figure 3:**
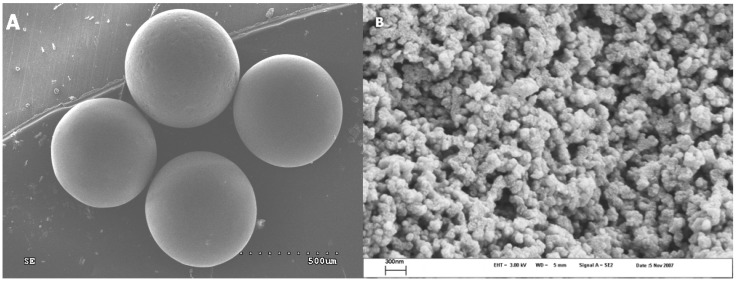
SEM micrograph of the SCN AC: (a) spherical particles forming the SCN. (b) ESEM micrograph of surface morphology of the SCN AC spherical particle.

Figure 3A shows a SEM micrograph of the PVPDVB-based SCN AC material; it indicates that the SCN AC spherical globules prepared in this study are uniform, with a size ~0.8 mm. As one can see from the ESEM micrograph (Figure 3B), the globules are made up of small (~60–90 nm) pseudospherical particles, forming aggregates. The SCN globules are approximately twice as large as those of the SKS [19]. Curved macropores ~0.15–0.5 µm in diameter are observed in the ESEM micrograph (Figure 3B), also being larger than those of the SKS. The macroscopic structure of the SCN AC according to the ESEM studies is disordered, and shows a little sign of self-similarity.

**Figure 4:**
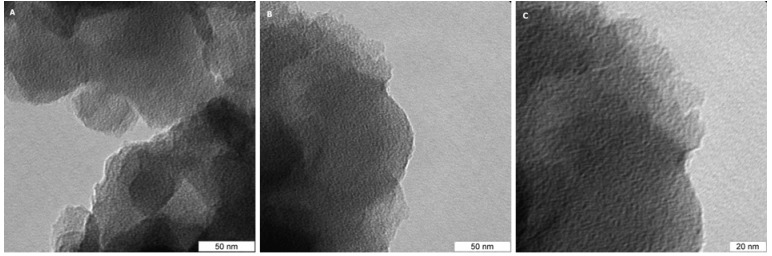
TEM micrographs of the SCN AC (a-c).

Figure 4 shows typical TEM micrographs of the PVPDVB-based SCN AC material recorded for different zones. No graphite-like regions are observed in any of the TEM micrographs. Although according to the results of the TEM examination the SCN AC material is nanostructured and nanoorganized at the molecular level with non-uniform particles of 2.5–5.0 nm in size, their aggregates possess no translation symmetry characteristic of graphene layers.

The SEM-EDX spectra (Table 1) indicate the main compositions of C and O on the SCN AC surface. The sensitivity of SEM-EDX is too low to register trace elements, in contrast to ESEM-EDX. ESEM-EDX data (Table 1) show the carbon composition of the SCN with small admixtures of residual oxygen, chlorine and sulfur (the presence of S and Cl in the sample is due to the precursor-resin preparation procedure, which involves chloromethylation and treatment of the copolymer with sulfuric acid [7]). Neither SEM-EDX nor ESEM-EDX detected any nitrogen in the outer surface layer of the SCN globules. No Fe-containing phases have been detected, contrary to the SKS AC [19].

**Table 1:** EDX results on the elemental composition of the SCN AC.

Element	SEM-EDX	ESEM-EDX	
	Exp.1	Exp.1	Exp.2
	Atom. %	Atom. %	Atom. %
C	94.60	89.71	89.8
O	5.40	9.97	9.89
N	-	-	-
S	-	0.16	0.17
Ca	-	-	-
Fe	-	-	-
Cl	-	0.15	0.14
Sum	100	100	100

### 2.2. Characterization on Atomic Level

Analysis of the QC simulated structures of the PVPDVB-based AC clusters (see Supplementary Materials for the Cartesian coordinates) along the carbonization process indicates a growing complexity and spreading of the structural characteristics of the clusters. It is obvious that a nanopore with a diameter *ca*. 3.5 Å forms in the bulk of the amorphous PVPDVB based AC. Also, a condensed polycyclic fragment, including five six-fold, two five-fold and one 7-fold ring with three N atoms, has been formed. This fragment is non-uniaxially curved, which confines the data on the graphene-organized domains in the ACs very well [21].

A non-uniform non-crystalline carbon 3D-lattice is formed upon dehydrogenation of the PVPDVB cluster under the cyclic boundary conditions. It should be strictly outlined that the 3D-lattice organized from the closed-loop clusters originally had no bonds between atoms in different cells. Therefore, the periodic cyclic boundary conditions can be applied to simulate the carbonization of oligomeric species, accompanied by crosslinking and transformation into bulk material.

The structure of the SCN simulated in the periodic model is amorphous and organized (one should note that periodicity of the AC does not imply crystallinity). Nanopores are clearly seen (Figure 2) both at boundaries between periodic units and inside the basic translated units. A condensed polycyclic fragment, including three six-fold, two five-fold, one 8-fold, one 4-fold and two 3-fold rings with 3 N atoms (plus one, attached as a nitrile – CN group to a phenyl residue) can be seen in periodic model #18. The polycyclic fragment is extremely curved, as in the case of the cluster model.

**Figure 5:**
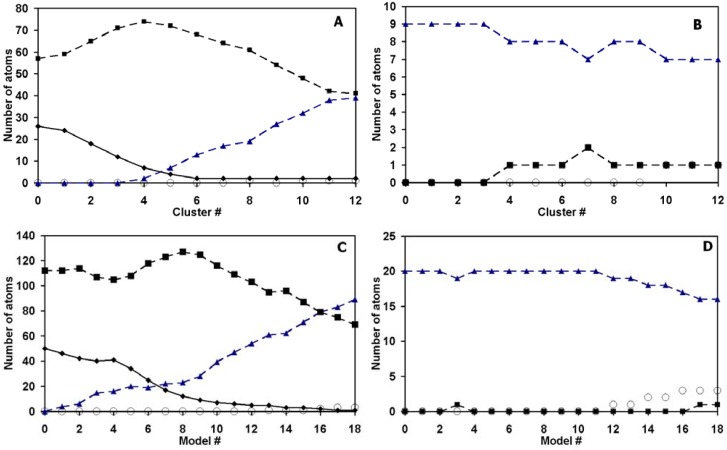
Change of the QC evaluated coordination number distribution for (a) carbon, (b) nitrogen atoms in the PVPDVB cluster and (c) carbon and (d) nitrogen atoms in the PVPDVB periodic model upon carbonisation. Solid line and rhombs – number of 4-fold coordinated atoms, dashed line and squares – number of 3-fold coordinated atoms, dashed line and triangles – number of 2-fold coordinated carbon atoms, circles – number of 1-fold coordinated carbon atoms.

The coordination number distribution for each cluster and periodic model in the series is shown in Figure 5. As one can see, trends in the coordination number distribution for clusters and periodic models correspond well. One should note that 1-fold coordinated atoms in the periodic models are artifacts, which correspond to termination of infinite carbonic chains in the framework of the model’s analysis.

The ring distribution is a valuable characteristic of the AC atomic structure, since it is closely related to the chemical properties of the carbon system. The starting PVPDVB cluster and periodic models only contain 6-fold rings, corresponding to the aromatic phenyl and pyridyl rings.

Both ring opening and ring formation upon carbon chain stacking have been observed in the QC simulation of the dehydrogenation of PVPDVB. The ring distribution has been evaluated for each cluster and each periodic model in the series. The dependence of the ring distribution on the H/(C+N) ratio is shown in Table 2 and Table 3.

**Table 2:** The dependence of the cycles distribution in cluster models on the H/(C+N) ratio.

Cluster #	H/(C+N) ratio	Cycle size/ number of cycles					
		3	4	5	6	7	8
0	0.924				11		
1	0.837	1		1	11		1
2	0.750	1		2	12		1
3	0.663	2	1	1	12		1
4	0.576	2	1	2	12		1
5	0.489	2		2	12		
6	0.403	2		2	12		
7	0.304	3	1	3	11	1	
8	0.228	2	1	3	12	1	
9	0.141	3	1	2	11	1	
10	0.076	3	1	2	10	1	
11	0.011	3	1	2	10	1	
12	0	3	1	2	10	1	

**Table 3:** The dependence of the cycles distribution in periodic models on the H/(C+N) ratio.

Model #	H/(C+N) ratio	Cycle size/ number of cycles					
		3	4	5	6	7	8
0	0.912				22		
1	0.868				22		
2	0.824	2			21		
3	0.769	2			21		
4	0.709	5	1		19		
5	0.659	6			18		
6	0.604	6	1		18		
7	0.555	6	1		18		
8	0.500	6	1		18		1
9	0.445	6	1	2	18		1
10	0.374	6	2	3	18		
11	0.313	5	3	3	19		
12	0.275	5	3	3	18		
13	0.214	6	3	3	18		
14	0.170	7	5	3	17		
15	0.110	7	5	2	17		
16	0.066	6	5	2	17		
17	0.033	6	5	4	16		
18	0	6	5	4	16		

It is also noteworthy that the shrinkage of the models (both cluster and translational) simulated upon dehydrogenation is clearly observed from the QC data.

As one can see, formation of pores and condensed polycyclic fragments, characteristic of the coordination number and ring size distributions demonstrate that the cluster and periodic models give analogous results. Therefore, they are consistent and can be used in order to study properties of the SCN on atomic (nano-) and bulk (micro-) levels in parallel.

### 2.3. Analysis of Experimental and Computational Data

The results of SEM, TEM and EDX measurements, along with statistical data of the QC evaluated model of the PVPDVB based AC allow us to characterize the SCN AC both at micro- and nano-levels. It should be pointed as a general conclusion that the differences in structural features between SKS [19] and SCN are minor and may be neglected to some extent. Hence, N-doping of the amorphous domain of ACs seems not to alter the structure.

The SCN AC consists of coagulated pseudospherical particles about 50 nm in diameter. Pores of non-uniform size and shape (100–150 nm in diameter) are formed between pseudospherical particles. As in the case of the SKS AC [19], the microstructure of the SCN AC directly reflects the history of formation, *i.e.* the precursor copolymer forms droplets in lyophilic media, which then dehydrogenate under heating without significant change of droplet-like morphology. This correlates well with the fact that one can regulate the microstructural parameters of the SCN AC by alteration of the biphase copolymerization conditions through change of the precursor droplets size [14].

As in the case of the SKS [19] no crystalline (graphite-like) phase is observed. So, in agreement with the conclusions of [22], the origin of the N-doped ACs macroscopic properties (*e.g.* catalytic activity, surface red/ox and acid/basic reactions, etc) should be sought in the amorphous domain structure.

It should be emphasized that no N has been detected on the outer surface layer of the SCN globules, despite the considerable nitrogen content in the bulk material [7]. This might mean that N is easily removed from the surface with volatile compounds during later synthetic procedures or storage, and, thus, does not govern chemical processes on the outer surface of the SCN globules.

One can say that the simulated structure of the amorphous domain of the SCN is characterized by a wide range of main features such as coordination numbers and ring distributions. One should note that there is a threshold in the dependence of these characteristics on the carbonization level. Both cluster and periodic models start only with 6-fold cycles and 3- and 4-fold coordinated backbone atoms. At the level of carbonization which refers to the H/(C+N) ≈ 0.7–0.5 these distributions begin to change, reflecting transformation of the polymer to the AC. Curved polycondensated fragments and pores of different nanodimensional size are observed. A set of active sites with different spatial surroundings of donor atoms and groups (usually N atoms and dehydrogenated benzene residues) is formed, providing a backbone for various physico-chemical processes, *e.g.* oxidation, chemi- and physico-sorption, *etc*. It is important that the N-active centers have different conformational environment, forming cavities of various sizes and, thus, enabling “host-guest” mechanisms of catalytic reactions, similarly to active centers of biological macromolecules.

Therefore, the cluster and periodic atomistic models of the SCN structure can be further used to study chemical and physical properties of the SCN AC in what concerns its amorphous domain (which seems to be dominating in this particular case of the AC material). Local processes should involve the cluster models, while the bulk ones can be studied with the periodic model.

## 3. Experimental and Simulation Section

### 3.1. Synthesis

The SCN AC has been manufactured by carbonization of PVPDVB copolymer by the method of [7], substantially similarly to that proposed in [14]. The initial resin contained up to 10% divinylbenzene as a crosslinking agent and had ionogenic groups containing methylpyidinium and pyridinic nitrogen. The carbonization of the PVPDVB copolymer has been carried out in two stages: in air at 623 K, and in Ar flow by a linear rise of the temperature to 1223 K (heating rate, 5 K/min) [7].

### 3.2. Electron Microscopy Characterization

Two models of electron microscope were used for examination of the SCN AC specimens by scanning electron microscopy (SEM). The SCN AC specimens, taken as is, were observed using a Jeol JSM50A scanning electron microscope operated at 15 kV. The elemental analysis used in the SEM (SEM-EDX) was performed by means of a Tracor-Northern energy dispersive X-ray (EDX) spectrometer mounted on the Jeol JSM50 A of the SCN AC specimens. The EDX detector was equipped with an ultra-thin light-element window detecting elements with an atomic number > 4.

The SCN AC specimens were observed using a Philips XL30 environmental scanning electron microscope (ESEM) operated at 3 kV were sputtered coated with a 4–8 nm layer of Au/Pt. The elemental analysis of the SCN AC specimens has been performed in the Philips XL30 ESEM integrated with a gaseous secondary electron detector and an EDX spectroscopy system (Philips EDAX-4 with a Si(Li) R-Super Ultra Thin Window detector). The SCN AC specimens were examined by the EDAX-4 system at an accelerating voltage of 20 kV with a counting time of 100 sec. The energy dispersive X-ray microanalysis used in the ESEM (ESEM-EDX) prevents problems with the sample charging and allows analyzing elemental compositions including the light elements such as carbon, nitrogen and oxygen.

Transmission electron microscopy (TEM) studies of the SCN AC were performed on a Zeiss 912 Omega microscope operated at 120 kV. Samples were suspended in acetone, sonificated for 10 min., deposited on carbon-coated Cu grids from acetone and dried for TEM studies.

### 3.3. Computational Methodology

The semiempirical calculations have been performed by QC method PM3 [26] with the “QuChem” program [23,27] for the cluster models and with MOPAC2007, kindly provided by Prof. J.J.P. Stewart [28,29], for the cyclic boundary conditions (periodic) models. The PM3 method is perfectly parameterized for reproducing spatial and electronic structure of the CH-containing compounds [30,31]. The restricted Hartree-Fock method has been applied for singlet (^1^*S*) state calculations of the clusters and periodic models. The complete space structure optimization with the evaluation of the wave function has been performed for each cluster and periodic model. Additionally, the structure of the periodic model has been optimized by cell parameters.

The cyclic boundary conditions model is better at describing the spatial structure and the processes occurring with the PVPDVB copolymer upon carbonization. However, the local chemical processes (*e.g.,* defects formation and genesis, or reaction of the AC surface with a small molecule like O_2_, H_2_O, etc.) cannot be studied using this approach. Moreover, the cyclic boundary conditions model is much more time-consuming. Therefore, it is reasonable to evaluate a cluster model of the same material, too. If the structural and energetic characteristics of the cyclic boundary conditions and cluster models are similar, one can use the latter one to study the local point processes, while the former one should be used to examine the bulk properties.

One should note that term “structure” has a different meaning from the conventional one in the case of amorphous solids. A sequence of translationally repeating units (crystal cells) is implied when a crystalline object is under study, with some possible defects. The structure is reduced to the spatial conformation of atoms in crystal cell. However, one don’t observe this in the case of amorphous matter – each volume, whenever small or big, contains unique numerical and spatial combination of atoms, which does not possess the property of translation through the solid. Therefore, the term “structure of amorphous object” needs another definition. Keeping in the mind that structural and electronic properties of amorphous matter are statistically distributed in a certain range [15], we propose to define this term as follows: structure of amorphous object is the spatial structure of certain volume (cluster) of the solid, which certain properties (previously chosen) lie within some statistical gap from the ones of any other volume of the same value. The reference volume is called *the model* of the amorphous solid, and it is said that this model is *adequate* to the real object.

## 4. Conclusions

The SCN AC has been characterized on micro- and nanoscale levels by SEM, TEM, EDX and QC simulations. The cluster and periodic models of the SCN have been QC evaluated. N-doping is been noticed to have a minor effect on the structure of the SCN amorphous domain. The studies performed indicate a great similarity in structures of the SKS and SCN ACs, allowing us to compare them directly. The cluster and periodic models of the SCN are consistent and might be used for further studies of chemical and physical properties of the SCN AC.

## Supplementary Data

Supplementary data can be downloaded online at http://www.mdpi.com/1996-1944/2/3/1239/s1/.
