# Supplementary material for: Characterization by SEM, TEM and Quantum-Chemical Simulations of the Spherical Carbon with Nitrogen (SCN) Active Carbon Produced by Thermal Decomposition of Poly(vinylpyridine-divinylbenzene) Copolymer
Source: Materials (Basel). 2009 Sep 7;2(3):1239–51. doi: 10.3390/ma2031239 (PMC5445732; doi:10.3390/ma2031239)
Supplement: Supplementary file 1 [file materials-02-01239-s001.zip › Supplementary Data/Description of Supplementary Data.pdf]

Characterization by SEM, TEM and Quantum-Chemical Simulations of the Spherical Carbon with Nitrogen (SCN) Active Carbon Produced by Thermal Decomposition of Poly(vinylpyridine-divinylbenzene) Copolymer

Volodymyr D. Khavryuchenko [1], Oleksiy V. Khavryuchenko [2,*], Andriy I. Shkilnyy [2,†], Denys A. Stratiichuk [3] and Vladyslav V. Lisnyak [2,*]

[1] Institute for Sorption and Problems of Endoecology, National Academy of Sciences of Ukraine / 13 General Naumov str., UA-03167, Kyiv, Ukraine; E-Mail: vkhavr@rumbler.ru

[2] Chemical Department, Kyiv Taras Shevchenko National University / 64 Volodymyrska str., UA-01033, Kyiv, Ukraine; E-Mail: andriy.shkilnyy@gmail.com

[3] Institute for Superhard Materials, National Academy of Science of Ukraine / 2 Avtozavodska str., UA-04074, Kyiv, Ukraine; E-Mail: strat1@yandex.ru

[†] Present address: Université de Tours, UFR de Sciences Pharmaceutiques / 37200 Tours, France

* Authors to whom correspondence should be addressed; E-Mails: alexk@univ.kiev.ua (O.V.K.); lisnyak@univ.kiev.ua (V.V.L.); Tel. +38-044-258-1241; Fax: +38-044-258-1241

Description of Supplementary Materials

Supplementary materials includes *.xyz files with the Cartesian coordinates of the cluster, optimized in ^{1}S RHF, basic units and supercell Cartesian coordinates of the models and the protocols of the computation *.out.

The name of the file consists of the system *NAME* (number of cluster – *C* or model *M*) and an addition, revealing the carbonization stage. The files like *NAMEX*.* (where $X = 0 – 12$) or *NAMEY*.* (where $Y = 0 – 18$) correspond to the number of the system.
